# An Innovative Transitional Care Unit for Hospital to Home Transition of Children With Medical Complexity: A Qualitative Study of Parents' Experiences

**DOI:** 10.1111/cch.70253

**Published:** 2026-03-05

**Authors:** Heleen N. Haspels, Nicole Skomorowski, Koen F. M. Joosten, Matthijs de Hoog, Job B. M. van Woensel, Clara D. van Karnebeek, Mattijs W. Alsem

**Affiliations:** ^1^ Department of Pediatric Intensive Care Amsterdam UMC, location AMC Amsterdam the Netherlands; ^2^ Amsterdam Reproduction and Development Research Institute Amsterdam the Netherlands; ^3^ Department of Neonatal & Pediatric Intensive Care, Division of Pediatric Intensive Care Erasmus Medical Center–Sophia Children's Hospital Rotterdam the Netherlands; ^4^ Transitional Care Unit Consortium the Netherlands; ^5^ Faculty of Medicine Amsterdam UMC, University of Amsterdam Amsterdam the Netherlands; ^6^ Departments of Pediatrics and Human Genetics, Emma Center for Personalized Medicine Amsterdam UMC, location AMC Amsterdam the Netherlands; ^7^ Department of Rehabilitation, Amsterdam Movement Sciences Amsterdam UMC, location AMC Amsterdam the Netherlands; ^8^ Department of Rehabilitation, Physical Therapy Science and Sports, UMC Utrecht Brain Center University Medical Center Utrecht Utrecht the Netherlands

**Keywords:** care innovation, children with medical complexity, hospital to home transition, qualitative research, transitional care unit

## Abstract

**Background:**

Hospital‐to‐home (H2H) transitions of children with medical complexity (CMC) are a multifaceted process with many challenges and obstacles, especially for parents. The ‘Jeroen Pit Huis’ (JPH) is a stand‐alone, innovative transitional care unit (TCU) that aims to improve the transition home. This study explored parents' experiences with the H2H transition via the JPH TCU setting, including the facilitators and barriers that shaped this process.

**Methods:**

A qualitative study using semistructured interviews was conducted between January and November 2023. Interviews were audio recorded and transcribed verbatim and analysed thematically. Data collection continued until thematic saturation was reached.

**Results:**

Twenty‐one parents (13 mothers and 8 fathers) of 14 CMC participated in 14 interviews. Inductive thematic analysis identified the following five interrelated processes shaping parental experiences: regaining control and confidence, emotional recovery and resilience, relational dynamics and family adaptation, navigating care systems, as well as child well‐being and development. Across these processes, parents reported key facilitators—grouped into the following four domains: TCU supportive environment, professional guidance and continuity, family and peer empowerment, as well as coordinated care systems. At the same time, barriers were described across four domains: privacy and boundaries, emotional and psychological strain, family equity challenges, as well as systemic and practical barriers.

**Conclusion:**

Parents experienced H2H transition via the TCU valuable for the well‐being and development of both their child and family. By capturing both facilitators and barriers, this study underscores the opportunities and challenges of a stand‐alone TCU and provides insights to inform the development of transitional care for CMC.

## Summary

- A stand‐alone transitional care unit offers a crucial bridge between hospital and home for children with medical complexity and their families, combining skill‐building with family adaptation.
- Parents gained confidence and preparedness for home care through supervised training, practicing emergencies and continuous professional presence in a homelike setting.
- Psychosocial support, family togetherness and peer connections fostered emotional recovery and resilience, emphasizing the importance of nontechnical aspects of transitional care.
- Four key facilitators—supportive environment, professional guidance and continuity, family and peer empowerment and coordinated care systems—shaped positive transitional care experiences.
- Barriers such as privacy concerns, emotional strain, family equity challenges and systemic obstacles underline the need for clearer communication, role clarity and stronger cross‐system coordination.

## 1 Introduction

Children with medical complexity (CMC) present a diverse array of children with complex chronic conditions, functional limitations, high healthcare use and substantial family‐identified service needs (Cohen et al. 2011). The care required for these patients extends beyond the confines of medical interventions; it encompasses a comprehensive and intensive approach that resonates through all aspects of family life. Parents of CMC are tasked not only being a parent who have to learn medical and nursing care for their child, but also to establish and maintain a care network around their child, requiring knowledge of the social support system and often to bear a considerable administrative burden (Seppänen et al. 2021; Ronan et al. 2020; Leyenaar et al. 2017). Additionally, the emotional toll of witnessing a child with complex chronic conditions can lead to heightened stress levels that interfere with parental mental health and impact family dynamics and child development (Peckham et al. 2014; Frankel et al. 2022).

For both parents and health care professionals, this may be extremely challenging as the complexity of care necessitates a high level of attention, support and coordination (Berry et al. 2014; Berry et al. 2013). Often CMC receive substandard care that is uncoordinated, fragmented and crisis driven (Allshouse et al. 2018; Arthur et al. 2018). This is reflected in poor health outcomes, high rates of emergency department visits and prolonged or recurrent hospital (re)admissions, as well as increased health‐care costs (Simon et al. 2010; Berry et al. 2011; Gold et al. 2016; Bramlett et al. 2009). To support CMC and their families, several complex care models have been developed, including primary care–centred ‘medical homes,’ tertiary care–based co‐management models and episode‐based approaches targeting specific transition points (Pordes et al. 2018). Episode‐based models include interventions targeting hospital‐to‐home (H2H) transitions, a particularly vulnerable period due in part to complex nursing tasks, a transfer of responsibility to parents and the need for coordination between hospital and community providers (van de Riet, Alsem, van der Leest, et al. 2023; Haspels, Knoester, et al. 2025). When H2H transitions are suboptimal, studies report medication discrepancies, gaps in home health care and caregivers feeling insufficiently prepared for complex home care; poor transitional care is also linked to unplanned post discharge reuse, including ED visits and readmissions (Auger et al. 2018; Coller et al. 2018; Nageswaran et al. 2020; Philips et al. 2019).

To improve H2H in the Netherlands, the Jeroen Pit Huis (JPH) was established (Het Jeroen Pit Huis 2022). Opened in April 2022 on the premises of Amsterdam University Medical Center (UMC), this transitional care unit (TCU) offers families separate, home‐like apartments where they live together while gradually adapting to their new reality and practising the complex care their child requires until they are ready for discharge. Length of stay ranges from approximately 1 week to 3 months, depending on child and family needs. A multidisciplinary team of nurses, family counsellors and paediatricians supports parents as they progressively assume the role of primary caregiver, guided by a structured care pathway (Supplementary 1; Table S1). By providing targeted preparation, coordination and support during this vulnerable H2H phase, the JPH model aims to improve child and family outcomes and reduce potentially avoidable health‐care utilization (Haspels, Mikkers, et al. 2025).

Despite growing interest in care models to support CMC, including TCUs, evidence on how parents experience care in these settings remains limited. Understanding parents' experiences, including perceived facilitators and barriers, is essential to inform continuous improvement of the JPH care pathway and to support the broader shift from practice‐based to evidence‐based transitional care. Moreover, these insights may generate transferable lessons for the international development and evaluation of similar TCUs. Therefore, the aims of this study were to explore parents' experiences with the JPH and to identify key facilitators and barriers during the hospital to home transition.

## 2 Methods

### 2.1 Study Design

A qualitative research design was utilized as this pragmatic approach is well‐suited to explore and describe the subjective experiences of receiving care in the JPH. This study adheres to the Standards for Reporting Qualitative Research (SRQR) checklist (Supplementary 2) (O'Brien et al. 2014).

### 2.2 Research Ethics

Approval was provided by the Institutional Review Board of the Amsterdam UMC, location AMC, who waived the need for a full ethical review (W22_410#22.485). Parental informed consent, including approval for audio recording, was obtained verbally before each interview.

### 2.3 Study Setting

The JPH is a stand‐alone TCU located on the premises of the Amsterdam UMC, the Netherlands. The TCU consists of eight fully equipped family apartments. Each apartment includes a separate bedroom for the CMC, a master bedroom for the parents, a living room, bathroom, kitchenette, a mezzanine level with a bed and desk for siblings if needed and a private outdoor area. In addition, the JPH offers several communal facilities, including a large kitchen, a shared living room with workspaces, areas for relaxation and play, a ‘snuggle room,’ an adapted bathroom for persons with disabilities, a physiotherapy gym and a garden with a playground.

In the JPH, at least one parent must be present 24/7, and siblings are also welcome to stay in the apartment. The TCU team consists of nurses, family counsellors and paediatricians. Additionally, we collaborate with other healthcare professionals, including allied health professionals, home care providers and pharmacy personnel in both the hospital and home settings. Nursing care and supervision of a dedicated paediatrician is available 24/7. During the stay, more and more responsibilities and tasks are gradually transferred to the parents until they become the primary caregivers. This process is guided by a newly developed structured care pathway, which is informed by the parental needs identified within our consortium (van de Riet, Alsem, Beijneveld, et al. 2023). This care pathway outlines the transition process for parents and children as they move from being care recipients to taking on the role of care providers and coordinating their own care. It is organized into seven phases, each with a step‐by‐step transition plan. See Supplementary 1 for additional information about the seven‐phase care path.

### 2.4 Study Population

The study population of the JPH consisted of children aged 0–18 years who were admitted to the hospital with a chronic complex condition and/or with an (expected) continuous dependence on medical technology after discharge. In addition, an anticipated need for specialized medical, nursing and/or allied health care following discharge was required. Admission to the JPH was indicated when discharge home was not yet feasible due to organizational, care‐related or family circumstances. Only children in a medically stable condition and/or with a set treatment regimen were eligible. The average stay ranges from a minimum of 2 weeks to a maximum of 3 months, depending on the needs of the child and family. The JPH primarily serves children from the Amsterdam UMC, although children can be referred from hospitals in or outside the Amsterdam region.

### 2.5 Participant Selection

Participants eligible for recruitment were parents who had stayed at the JPH. A parent was defined as a person who served in a primary caregiver role and provided constant and sustained care to the child during their stay in the JPH. Inclusion criteria encompassed adults (≥ 18 years) with a minimum 1‐week stay at the JPH, having returned home at least 2 weeks after their stay in the JPH and proficiency in Dutch or English.

We applied purposive sampling to ensure heterogeneity in the study sample. Selection was based on child characteristics (e.g., diagnosis, technology dependence), length of stay in the JPH, time since discharge, parental gender and social economic background. This approach facilitated the inclusion of a wide range of perspectives, enriching the research with comprehensive and varied data sources (Etikan et al. 2016). Eligible parents were first identified by the paediatrician of the JPH, who asked parents for permission to be contacted for the study. Subsequently, the research team contacted these parents by telephone to provide information about the study and to invite them to participate.

### 2.6 Data Collection

Semistructured, open‐ended interviews of approximately 1 h were conducted between January 2023 and November 2023. The lead interviewer was a medical master's student (N.S.), originally trained as a speech and language therapist with extensive experience in special education, but with no prior experience in qualitative research. The interviews were therefore supervised by a fourth‐year PhD student in paediatrics with specific expertise in hospital to home transitions for CMC (H.N.H.). H.N.H. has been formally trained in qualitative research methods and has prior experience conducting qualitative studies (Haspels et al. 2023). Neither interviewer was part of the patients' medical team. All interviews were audio‐recorded and field notes were made.

To maximize the range of perspectives and reduce participation barriers, such as distance from the hospital and the daily demands of caregiving, interviews were conducted based on the participants' preferences, either in person at the JPH, at their homes, or via teleconference.

To ensure that questions were understandable for parents, the interview guide was pilot‐tested with a CMC parent (see Supplementary 3). The guide covered experiences during the hospital stay, the transition to and stay at the JPH, and the transition home and invited parents to reflect on both what helped and what could be improved. Predesigned questions and follow‐up prompts were used to explore parents' perspectives, including perceived facilitators and barriers in the transition home. Demographic data from parents were obtained at the end of the interview including their age, gender, living status, education and occupational level, language spoken at home, as well as their child's gender, age, medical diagnosis, technological support at home and home care arrangements. Data saturation was established when two consecutive interviews failed to provide new and relevant information (Morse 1995).

### 2.7 Data Analysis

Demographic and clinical characteristics of the participating children and families were summarized using descriptive statistics, with categorical variables reported as *n* (%) and continuous variables as medians with ranges. Interviews were transcribed verbatim and all identifying information was removed. Data were analysed using inductive thematic analysis (Braun and Clarke 2006), facilitated by qualitative data analysis software (MAXQDA). The analysis followed an iterative process: First, transcripts were read multiple times to ensure familiarity with the data. Next, initial codes were generated and systematically applied across transcripts. Codes were then reviewed, compared and clustered into broader categories, and overarching themes were developed to capture parents' experiences. Within these themes, we identified codes describing facilitators and barriers. In a final analytic step, these facilitator and barrier codes were examined across themes and inductively grouped into cross‐cutting domains. Transcripts were independently coded by two investigators (N.S. and H.H.), who then compared and discussed their coding until consensus was reached, thereby ensuring dependability through investigator triangulation. Reliability was further enhanced by a third‐party review (M.A.), who critically examined the coding framework and emerging themes. Following data analysis, member checking of results with parents was completed to ensure credibility of findings. A summary of the identified themes was shared online with all participants, who were invited to provide feedback in case they disagreed with the findings. No changes to the results were made as no participants expressed disagreement.

## 3 Results

### 3.1 Participants

In total, 21 parents (8 fathers, 13 mothers) of 14 families who stayed at the JPH participated in the study. Eight parents did not consent; three felt overwhelmed, and five did not find time. In two instances, patients did not transition home but first underwent a transition to the hospital for surgery. Two families had been admitted to the TCU twice. Once as a ‘step‐down’ function in the transition from hospital to home, and once as part of a ‘step‐up’ function with an admission directly from home. The interviews were conducted at a median time of 3.4 months (range 1–7.2) after (last) JPH discharge. Characteristics of children and parents are shown in Tables 1 and 2.

**TABLE 1:** Characteristics of children admitted to the Jeroen Pit Huis (JPH).

	Total *N* = 14
Female	7 (50)
Median age in months	8 (0.7–180)
Median hospital LOS prior to JPH stay in months	1 (0.2–4)
Median JPH LOS in months	1.6 (0.2–6)
Diagnoses	
Meningitis by hydrocephalus	1 (7.1)
Schaaf–Yang syndrome	1 (7.1)
Stickler syndrome	1 (7.1)
Pierre Robin sequence	1 (7.1)
Crouzon syndrome	2 (14.3)
Cystic gibrosis	1 (7.1)
Trisomy 21 with AVSD	1 (7.1)
Prader‐Willi syndrome	1 (7.1)
Multiple congenital anomalies	1 (7.1)
Perinatal asphyxia	1 (7.1)
Oesophageal atresia	1 (7.1)
Malan syndrome	1 (7.1)
Spina bifida	1 (7.1)
Amount of technology at home	1 (0–4)
Type of technology at home	
Nasogastric tube	9 (64.3)
Nebulizer	2 (14.3)
Oxygen therapy/mechanical ventilation	2 (14.3)
Tracheostomy	1 (7.1)
Nasopharyngeal tube	1 (7.1)
Monitor	1 (7.1)
Central line	1 (7.1)
Suction device	1 (7.1)
Home care	10 (71.4)
Amount of total hospital admissions	
1 time	7 (50)
2 times	3 (21.4)
6 times	2 (14.3)
10 times	1 (7.1)
More than 40 times	1 (7.1)

*Note:* This table presents demographic and clinical characteristics of the study population, including sex, age, hospital and JPH length of stay, diagnoses, technology dependence, home care use and number of prior hospital admissions. Data are shown as *n* (%) or median (range).

Abbreviations: AVSD, atrioventricular septal defect; JPH, Jeroen Pit Huis; LOS, length of stay.

**TABLE 2:** Family demographics of participants in the study.

Number of interviews/families	14 (100)
Interviews at the JPH	6 (42.9)
Interviews at home	5 (35.7)
Online interviews	3 (21.4)
Participants	21 (100)
Male	8 (38)
Median age of parents in years	37 (27–73)
Country of birth	
Netherlands	13 (61.9)
Iran	2 (9.5)
Congo	1 (4.8)
China	1 (4.8)
Turkey	1 (4.8)
France	1 (4.8)
Morocco	2 (9.5)
Dutch speaking	18 (85.7)
Home language	
Dutch	9 (64.3)
Farsi	1 (7.1)
Chinese	1 (7.1)
English	1 (7.1)
Moroccan	1 (7.1)
Turkish	1 (7.1)
Parents living together	11 (78.6)
Educational level^a^	
Low	5 (23.8)
Medium	6 (28.6)
High	10 (47.6)
Occupational level^b^	
Level 1	7 (33.3)
Level 2	7 (33.3)
Level 3	1 (4.8)
Level 4	6 (28.6)
Amount of children in the family	1.5 (1–6)
Ranking index child in the family	
Only child	7 (50)
Oldest of the 2 children	1 (7.1)
Youngest of the 2 children	3 (21.4)
Youngest of the 3 children	2 (14.3)
Fourth of the 6 children	1 (7.1)

*Note:* This table describes the demographic characteristics of participating families and parents, including interview setting, parent age, country of birth, home language, family composition, educational level, occupational level and ranking of the index child within the family. Data are *n* (%) or median (range).

^a^ Educational level of all interviewed parents based on CBS data (statline.cbs.nl).

^b^ Occupational level of all interviewed parents categorized according to the ISCO system for professions (http://www.ilo.org/public/english/bureau/stat/isco).

### 3.2 Themes

The inductive thematic analysis resulted in the following five interrelated themes that capture parents' experiences of transitioning from hospital to home through the TCU: (1) regaining control and confidence, (2) emotional recovery and resilience, (3) relational dynamics and family adaptation, (4) navigating care systems and (5) child well‐being and development. Each theme describes the processes parents went through, as well as the facilitators and barriers that shaped these experiences (see Table 3 for an overview of the results).

**TABLE 3:** Themes, facilitators, barriers and illustrative quotes of parents' experiences with transitional care at the Jeroen Pit Huis (JPH).

Theme	Focus (process)	Facilitators	Barriers	Illustrative quotes
**1. Regaining control and confidence**	Parents moved from dependence on professionals towards regaining control and confidence in caring for their child.	Collaborative decision‐making Private apartments Homelike environment Structured training 24/7 together as family 24/7 nurses	Frequent professional visits Initial fear and uncertainty with technical caregiving tasks	*In the hospital, they just come in without asking. Even if you put a sign on the window saying ‘he's sleeping’. They still come in because it's medication time. And at the JPH, they just knock politely, ask if it's okay to come in at a certain time. That gives you a sense of privacy again, a bit of control. (Parent K)* *You start with some simpler tasks, in this case, it was suctioning the nasopharyngeal tube. Clearing it of mucus, snot, food, milk residues. First, you begin with that, after doing it so many times, then you move on to the next step. That's how we have taken each step gradually. First, observe, then do it yourself once, then do it yourself several times, with one of the nurses observing, and then three times completely independently. And that's how we have addressed each step. (Parent C)* *And then, in the last week, we also did the nights ourselves. And that's when we started to get used to it, well, okay, let us say if we were to go home for a day, how would that be for us? And do we feel comfortable enough to be alone at home for a night because we experience the days here, but it's different at night. Suppose something happens, how do you anticipate that? (Parent B)*
**2. Emotional recovery and resilience**	Parents needed time and space to process suppressed emotions after hospital stays and traumatic experiences.	Private apartments 24/7 together as family 24/7 nurses Proximity to hospital Access to psychosocial care workers and family counsellors Homelike environment	Difficulty accessing emotions after cumulative traumatic experiences Feeling overwhelmed	*We were finally able to get some rest; she (the mother) was broken down both mentally and physically*. *During the stay at the JPH, I believe there has been a lot of grief. I have also reflected a lot on the breastfeeding bond that I had envisioned. It was completely disappointing, taken away, and initially, I felt that our child had no bond with us. That still makes me very sad, even now. (Parent G)*
**3. Relational dynamics and family adaptation**	Relational dynamics within the TCU—spanning professional, peer and family relationships—were crucial in supporting adaptation to a new family life at home.	Private apartments Homelike environment 24/7 together as family Recognition by nurses, doctors and facility staff Attention to siblings by professionals Peer‐to‐peer support Peer companionship for siblings	Blurred boundaries Initial sibling distress Lack of uniform policy	*Really, they are there for you. They come to you to ask how they can help and how you are feeling. They make you feel like you are not alone. (Parent F)* *It was just beautiful to see that I gained confidence through them (other families) or something. My little one had to undergo surgery, and I overheard a mother who had gone through the same thing. I had already been through so much, but through them, I found strength. (Parent M)* *Her father was suddenly away for 2 months in the hospital with her brother. I was at home with her little sister, who was 1 year old. (...) She had nightmares, so every night she would wake up and start crying, calling for dad and her brother. That was a big problem for us, but then we started with the Jeroen Pit house, where we could go to them every evening and sometimes even stay. That was really good news for her. (Parent A)*
**4. Navigating care systems**	Care coordination across hospital, TCU, and home was a central aspect of parents' experiences, shaped by both supportive structures and communication challenges.	Multidisciplinary approach Support from TCU staff in arranging practical matters (home care, equipment, medication) Expertise and reliability of TCU staff as safety net	Lack of clear plan before hospital to TCU transfer Fragmented information exchange between hospital, TCU and home care services Parents feeling responsible for coordinating across systems Practical barriers in arranging external services and resources (e.g., equipment, medication, home care, day‐care placement, funding)	*It's also nice in the hospital nowadays; you have those multidisciplinary meetings, so you have the nursing staff, the doctors, and everyone together. We thoroughly discussed how we would approach things at the JPH. It's also very nice to be there as a parent, then you know exactly what's being discussed. And then we went to the JPH. (Parent E)* *The communication, and especially coordination, between the hospital and Jeroen Pit needs to improve. If you say we will see when you can go there, then do it. I understand that there may be panic about bed availability, but that cannot be conveyed to the people. (Parent C)* *Many things have been discussed. For example, we talked about him going to a medical daycare facility. But there is currently no space, so there is no care available. Therefore, I cannot work. The personal budget (funding) is not yet arranged. So practically speaking, I cannot go anywhere. Much has been talked about. However, when there are issues where you depend on a third party, you can discuss them, but it does not get resolved. (Parent I)*
**5. Supporting child development**	The TCU environment created opportunities for children's comfort and developmental progress that were less feasible during hospital admission.	Homelike environment Indoor and outdoor play facilities Opportunities for social interaction with siblings and peers Freedom of movement between rooms and outdoor areas	—	*Our daughter just thrived there due to all the attention she received and the space. Despite having a lot of sadness as well. (Parent D)* *And I think the biggest difference was that he was more mobile; he could walk from the bedroom to the living room, to the toilet, or to the corridor, which he could not do in the hospital. He quickly regained his muscles. In the hospital, he was always weak, and the doctor said he must be mobile, he must walk, but in the hospital, it wasn't possible. It was easier at Jeroen Pit. (Parent A)*

*Note:* This table presents five overarching themes describing parents' experiences with transitional care at the JPH. For each theme, the main focus or process is outlined, along with identified facilitators and barriers. Illustrative quotes are included to exemplify parental perspectives and provide contextual depth.

#### 3.2.1 Theme 1: Regaining Control and Confidence

A central process described by parents was the gradual shift from dependence on professionals to regaining a sense of control and confidence in caring for their child. In the hospital, many parents felt restricted, with limited autonomy over daily routines and care decisions, often experiencing that care was imposed on them rather than discussed. At the JPH, this shifted towards a more collaborative approach in which parents were actively involved in seeking solutions together with professionals. The transition towards self‐reliance was further facilitated by the private apartment setting, where parents determined who entered their space and organized daily life according to their own rhythm. At the same time, some parents noted that the frequent visits from healthcare professionals, although intended to provide support, could feel overwhelming and occasionally limited the sense of privacy and control. The rural, spacious and homelike environment, both indoors and outdoors, further reinforced this sense of freedom compared to the relatively small hospital rooms. This freedom enabled parents to regain control over daily routines and to undertake other tasks, such as work‐related matters, which they viewed as a positive and empowering step.

In parallel, parents learned to take on complex caregiving tasks through structured, step‐by‐step training. Initially, performing technical procedures was perceived as daunting, accompanied by fear and uncertainty. However, the continuous 24/7 presence of nurses under paediatric supervision created a safe environment to practice and gradually increase responsibilities. Importantly, this also included practicing night‐time care, which parents identified as a critical aspect of building confidence for life at home. Both parents were able to participate in caregiving, allowing them to share experiences, practice tasks together and develop a joint sense of competence. Certification of newly acquired skills and repeated opportunities to manage emergencies under supervision were particularly important in fostering parental competence. Over time, these experiences translated into growing confidence and a sense of preparedness to take full responsibility at home.

#### 3.2.2 Theme 2: Emotional Recovery and Resilience

Parents described a broad spectrum of emotions during their transition, ranging from stress, anxiety and uncertainty about the future to feelings of guilt, shame and grief. Many had undergone traumatic experiences, such as complicated childbirth or intensive care admissions, and reported suppressing their emotions during hospitalization in order to ‘stay strong.’ Upon arrival at the TCU, their first need was often to settle emotionally after these demanding experiences. This recovery was facilitated by the opportunity to rest in a comfortable apartment with their child, while nurses temporarily assumed responsibility for care—particularly at night. Being together again as a family, without the constant commute between hospital and home, brought relief for both parents and siblings. The rural, spacious and homelike environment—both indoors and outdoors—further helped restore a sense of freedom and inner peace, positively influencing parents' well‐being compared to the relatively small and restrictive hospital rooms. The setting was also perceived as safe due to the proximity of nurses and doctors and the location next to the hospital, which contributed to peace of mind. These conditions allowed space for a wider range of emotions to surface: Some parents became tearful, and others experienced irritability or anger. Round‐the‐clock support from nurses, alongside access to psychosocial care workers and family counsellors, created outlets for these emotions and fostered resilience over time. At the same time, the process was not without challenges. Several parents emphasized that the accumulation of traumatic experiences could make it difficult to access or articulate their emotions, and some described feeling temporarily overwhelmed once they finally had space to reflect.

#### 3.2.3 Theme 3: Relational Dynamics and Family Adaptation

Relational dynamics within the TCU—spanning professional, peer and family relationships—were crucial in supporting adaptation to a new family life at home. Parents often emphasized their interactions with healthcare professionals as an important source of support. Unlike in the hospital, where attention was primarily directed towards the ill child, the JPH created space for the whole family to be seen and acknowledged. Parents felt recognized not only by nurses and doctors but also by facility staff, who gave attention to siblings by offering distractions during care moments or explaining procedures in child‐friendly ways. While these gestures were mostly appreciated, some parents found it intrusive when staff occasionally assumed a parental role with siblings inside the family's apartment. Connections with other families were also described as highly valuable. Parents appreciated the mutual understanding that arose from sharing similar challenges, and siblings found companionship and distraction through contact with peers in comparable situations. At the same time, close proximity sometimes created tensions; some parents perceived disparities in how families were treated and expressed a need for a more uniform policy. Finally, many parents highlighted the importance of reconnecting within their own family. After long periods of separation during hospitalizations, being together again under one roof fostered a sense of unity and normality. The opportunity to share caregiving responsibilities and to stay together overnight was described as particularly meaningful. Siblings, who initially showed signs of stress or withdrawal, became more at ease in the homelike environment, with some regaining confidence and joy as they reintegrated into daily family routines.

#### 3.2.4 Theme 4: Navigating Care Systems

Care coordination across hospital, TCU and home was a central aspect of parents' experiences, shaped by both supportive structures and persistent communication challenges. Parents valued the multidisciplinary approach that connected hospital teams with TCU staff. Preparatory meetings before transfer were seen as particularly helpful, as they provided clarity and facilitated the development of a comprehensive care plan. When such a plan was missing, however, parents described uncertainty and confusion, not only for themselves but sometimes also among professionals involved. Clear and consistent communication about transfer procedures and expectations was considered essential, yet not always achieved. Positive examples included the involvement of professionals such as the rehabilitation physician, who maintained continuity with the medical day‐care facility, and pre‐established arrangements with general practitioners, which were highly appreciated. Parents also highlighted the supportive role of TCU staff in navigating practical issues, such as arranging home care, medical equipment or medication. The expertise and reliability of staff were experienced as a safety net that extended even after discharge. At the same time, challenges remained. Exchanging information across the hospital, TCU and home care services was often fragmented, leaving parents feeling responsible for coordinating between different systems. Practical barriers in arranging external services and resources—such as equipment, medication, day‐care placement or funding—were frequently mentioned. Parents emphasized that while many of these issues were discussed, they could not always be resolved when dependent on third parties, which limited their ability to return to daily life, including work. These challenges left parents feeling responsible for coordination and struggling to resolve issues when dependent on third parties.

#### 3.2.5 Theme 5: Child Well‐Being and Development

Parents observed that the TCU environment created opportunities for their child's comfort and developmental progress that were less feasible during hospital admission. They described how the homelike atmosphere, indoor and outdoor play facilities, as well as opportunities for social interaction with siblings or other children contributed to a more stimulating environment. This setting was perceived as supportive for both emotional well‐being and psychomotor development. Parents highlighted that older children, in particular, gained physical independence more rapidly than in the hospital, as they were free to move between rooms and outdoor areas instead of being confined to a hospital bed or ward. Physiotherapy exercises were also easier to integrate into daily life at the TCU, allowing children to practice skills more naturally within their living environment.

### 3.3 Facilitators and Barriers

Across all themes, parents described a set of cross‐cutting facilitators and barriers that shaped their experiences of transitional care at the JPH. Facilitators clustered into the following four domains: a supportive environment, professional guidance and continuity, family and peer empowerment, as well as coordinated care systems. In contrast, barriers emerged across the following four domains: privacy and boundaries, emotional and psychological strain, family dynamics and equity challenges, as well as systemic and practical barriers. An overview of these domains and their associated factors is presented in Table 4.

**TABLE 4:** Domains of facilitators (+) and barriers (−) in parents' experiences of transitional care at the Jeroen Pit Huis (JPH).

Facilitator domains (+)	Key facilitators
**TCU supportive environment**	Private apartments; homelike environment; proximity to hospital
**Professional guidance and continuity**	24/7 nurses/professional presence; structured training; access to psychosocial care workers and family counsellors; recognition by nurses, doctors and facility staff; attention to siblings by professionals
**Family and peer empowerment**	24/7 together as family; collaborative decision‐making; peer‐to‐peer support; peer companionship for siblings
**Coordinated care systems**	Multidisciplinary approach; support from TCU staff in arranging practical matters; expertise and reliability of TCU staff as safety net

Barrier domains (−)	Key barriers
**Privacy and boundaries**	Frequent professional visits limiting privacy; blurred professional–parental roles with siblings
**Emotional and psychological strain**	Initial fear and uncertainty with technical tasks; difficulty accessing emotions after cumulative traumatic experiences; feeling temporarily overwhelmed once space to reflect emerged
**Family dynamics and equity challenges**	Initial sibling distress; perceived unequal treatment or lack of uniform policy between families
**Systemic and practical barriers**	Lack of clear care plan before hospital–TCU transfer; fragmented information exchange across hospital–TCU–home; parents feeling responsible for coordination; practical barriers in arranging external services (equipment, medication, day care, funding)

*Note:* This table summarizes the domains of facilitators and barriers reported by parents during transitional care at the JPH. Four domains of facilitators (+) highlight supportive structures and processes contributing to positive experiences, while four domains of barriers (−) illustrate challenges at the individual, family and system levels.

## 4 Discussion

This qualitative study provides insights into parents' perceived experiences at the innovative TCU JPH in the Netherlands. Thematic analysis identified the following five interrelated processes: regaining control and confidence, emotional recovery and resilience, relational dynamics and family adaptation, navigating care systems, as well as child well‐being and development. Together, these findings show that transitional care is a multilevel process, shaped by parent, family, system and child factors, and not solely by technical skill acquisition.

Consistent with prior qualitative work on direct hospital to home discharge, parents described difficulties with emotional processing and feeling unprepared for the transition (van de Riet, Alsem, Beijneveld, et al. 2023). Our findings suggest that the TCU context can mitigate this by helping parents **regain control and confidence** through structured training, supervised rehearsal of emergencies and 24/7 professional back‐up. Accordingly, and in contrast to earlier reports of low confidence with equipment use or emergency preparedness (Desai et al. 2016; Murdoch and Franck 2012; Amar‐Dolan et al. 2020), parents described feeling better equipped after practicing within the safety net of the TCU. This regained sense of safety also appeared to create room for **emotional recovery and resilience.** Parents described needing peace and security before they could process stress, grief and uncertainty. During hospital admission, many operate in ‘survival mode,’ which can hinder learning and information processing and increase overload, particularly given elevated anxiety and depression among parents of CMC (Bayer et al. 2021; Cohn et al. 2020). In this context, the perceived value of psychosocial guidance resonates with evidence that sustained self‐management under stress is difficult and that caregiver capacity should be supported as a shared responsibility rather than assumed as an individual task (Wong Chung et al. 2020).

Beyond individual parents, our findings also emphasize **relational dynamics and family adaptation,** showing that transitional care operates at the level of the wider family system. Siblings of children with long term conditions are often overlooked and are at increased risk of psychosocial impacts, which can add pressure on parents and family functioning (Blamires et al. 2024). Supporting the family unit therefore matters, as stronger family functioning is associated with better well ‐being of children with chronic conditions and may contribute to sustainable home care (Leeman et al. 2016).

On a more system level, **navigating care systems** showed that gains in parental confidence can be undermined when coordination across hospital, TCU and community services remains fragmented. Parents valued structured handovers and a shared care plan, which echoes earlier work showing that families prioritize clear roles, anticipatory planning and reliable communication during hospital to home transitions (Desai et al. 2016). However, consistent with prior studies, parents still described feeling like the central ‘hub’ responsible for bridging information gaps and arranging services, particularly when home health care, equipment or other external resources depended on third parties (Nageswaran et al. 2020). These findings reinforce that transitional care requires not only within unit preparation, but also robust cross setting communication mechanisms and shared accountability to reduce caregiver burden and support sustainable care at home.

Regarding **child well‐being and development**, our findings align with Price et al., who reported that once technology dependent children are medically stable, both parents and professionals perceive the hospital as a suboptimal setting that can hinder child well‐being and development (Price et al. 2018). This resonates with evidence that CMC admitted for prolonged hospitalization often have delayed gross motor development and slower acquisition of gross motor skills during admission (Pflock et al. 2023). In our study, parents similarly described how the homelike TCU environment enabled more mobility, more natural integration of physiotherapy into daily routines and perceived gains in physical independence, particularly for older children.

### 4.1 Essential Facilitators of TCU Care Across Themes

Across themes, parents' experiences were shaped by the following four recurring facilitators: a supportive environment, professional guidance and continuity, family and peer empowerment, as well as coordinated care systems. The value of a less clinical, homelike environment aligns with prior work showing benefits for family well‐being and confidence (Price et al. 2018). The importance of continuity and strong parent–provider relationships is consistent with complex care literature (Golden and Nageswaran 2012; Miller et al. 2009; Adams et al. 2017; Adams et al. 2013). Family and peer connectedness resonates with evidence that siblings are at risk of being overlooked during prolonged hospital‐based care (Frankel et al. 2022; van de Riet, Alsem, Beijneveld, et al. 2023; Deavin et al. 2018; Giallo et al. 2014). Finally, coordinated transitional care has been associated with reduced hospital days and improved family outcomes (Berman et al. 2005; Simon et al. 2012; Cohen et al. 2012).

### 4.2 Barriers and Improvement Targets

Despite the benefits of the TCU, parents described barriers that could constrain their transitional care experience. Concerns about privacy and blurred boundaries point to the need for clearer negotiation of roles and expectations, which has also been identified as a recurrent challenge in complex paediatric care and home care contexts (Kirk 2001; Petosa 2018). Emotional and psychological strain, including feeling overwhelmed once there was space to reflect, is consistent with evidence of elevated psychological distress among parents of CMC (Cohn et al. 2020). This underscores the importance for health care professionals to proactively recognize signs of overload and to normalize and facilitate timely psychosocial support and follow‐up, rather than assuming parents will cope independently. Parents also reported perceived inconsistencies between families, suggesting that transparent and uniform policies may be needed to support trust and equity. Finally, persistent fragmentation in cross‐setting communication and reliance on third parties for home care, equipment, medication or funding echoed prior findings that inconsistent communication increases uncertainty and caregiver burden and can contribute to safety risks (van de Riet, Alsem, van der Leest, et al. 2023; van de Riet, Alsem, Beijneveld, et al. 2023; Leonard et al. 2004; Adams et al. 2021).

### 4.3 Limitations

Data were collected at a single TCU, which may limit transferability. However, it is important to recognize that the concept of TCU is a new aspect of care. The detailed description of the setting and care model supports analytic transferability to similar transitional care contexts. Another limitation of our study is that admission history in the TCU was not a sampling criterion, and we cannot exclude that parents with repeated TCU admissions may describe different experiences. Finally, a limitation of our study is its exclusive focus on the parental perspective. While parents' experiences are essential, they do not provide the full picture. Siblings emerged as important actors, yet their voices were not included. Future studies should therefore also capture siblings' perspective to achieve a more comprehensive understanding of transitional care in the JPH.

### 4.4 Relevance to Clinical Practice

With the growing population of CMC and increasing emphasis on long term care, it is essential to critically examine how H2H transitions are organized (van de Riet et al. 2022; Boerman et al. 2023). This study suggests that a homelike TCU may support safer, more sustainable transitions through structured parent preparation and cross setting coordination, while highlighting improvement targets such as role clarity and boundaries, consistent communication and follow‐up psychosocial support after discharge. However, a TCU will not be necessary for all families; selection should consider caregiver readiness, existing home care capacity and broader family and social circumstances. The BRIDGE study is underway to quantify the effectiveness of the TCU model compared with direct discharge, using outcomes such as caregiver stress and self‐confidence, quality of life, family functioning and health care utilization (Haspels, Mikkers, et al. 2025; Haspels et al. 2023).

### 4.5 Conclusion

In conclusion, families experienced transition through the TCU JPH valuable for the overall well‐being and development of both the child and family. Their accounts show that effective transitional care goes beyond technical skill training and also depends on supportive conditions that help families adapt emotionally, relationally and practically. These insights can guide further refinement of TCU care and support the development of similar models.

## Author Contributions

All authors have approved the submitted version of the manuscript and agreed to be personally accountable for the authors' own contributions.

**Heleen N. Haspels:** conceptualization, investigation, methodology, writing – original draft, visualization, project administration, formal analysis, data curation. **Nicole Skomorowski:** conceptualization, investigation, data curation, formal analysis, writing – review and editing, visualization. **Koen F. M. Joosten:** conceptualization, writing – review and editing, supervision, funding acquisition. **Matthijs de Hoog:** conceptualization, writing – review and editing, supervision, funding acquisition. **Clara D. van Karnebeek:** conceptualization, writing – review and editing, supervision, funding acquisition. **Mattijs W. Alsem:** conceptualization, methodology, investigation, data curation, writing – review and editing, supervision, project administration. All authors have made substantial contributions to the conception of this work.

## Funding

This project is made possible by the Netherlands Organization for Health Research and Development (ZonMW; 845008701). The funder had no role in the design and conduct of the study.

## Ethics Statement

The study protocol was approved by the Medical Ethics Review Committee of the Academic Medical Center in Amsterdam, the Netherlands, which confirmed that the Medical Research Involving Human Subjects Act did not apply and the study was therefore compliant with the 1975 Declaration of Helsinki (Reference Number W22_410#22.485). The participants were informed that their participation was voluntary and that they had the option to withdraw from the study at any time.

## Consent

All participants consented verbally to their participation and the publication of the anonymized results prior to the audio recording.

## Supporting information


**Table S1:** Description of seven‐step transitional care pathway at the Jeroen Pit Huis.
**Supplementary 1:** Seven‐step care pathway in the Jeroen Pit Huis.
**Supplementary 2:** Standards for Reporting Qualitative Research (SRQR).*
**Supplementary 3:** Interview guide.

## Data Availability

The data that support the findings of this study are available on request from the corresponding author. The data are not publicly available due to privacy or ethical restrictions.
