# Supplementary material for: An Innovative Transitional Care Unit for Hospital to Home Transition of Children With Medical Complexity: A Qualitative Study of Parents' Experiences
Source: Child Care Health Dev. 2026 Mar 5;52(2):e70253. doi: 10.1111/cch.70253 (PMC12962879; doi:10.1111/cch.70253)
Supplement: Supplementary file 1 — Table S1: Description of seven‐step transitional care pathway at the Jeroen Pit Huis. Supplementary 1: Seven‐step care pathway in the Jeroen Pit Huis. Supplementary 2: Standards for Reporting Qualitative Research (SRQR).* Supplementary 3: Interview guide. [file CCH-52-e70253-s001.docx]

**Supplementary 1: Seven step care pathway in the Jeroen Pit Huis**

At the Jeroen Pit Huis (JPH), a seven step care pathway was developed prior to its opening, informed by best practices and existing literature (1). The model describes how parents and children gradually shift from being care recipients to becoming care providers and coordinators of the care process. In addition, the pathway was shaped by parental experiences and needs identified in a national consortium study, ensuring close alignment with family perspectives (2). Step-by-step transition plans with treatment goals are developed based on the needs of the child and parents, enabling a successful transition back home. In its current form—24/7 parental stay combined with direct nursing support under the supervision of pediatricians—the JPH is unique in the Netherlands.

Personalized treatment goals are established based on the specific needs of both the child and parents. These may vary per family but can generally be categorized under the domains of physical, financial, psychological, social, spiritual, and practical aspects, such as medical devices, legal considerations, and future or developmental issues.

An overview of the seven-step care pathway is presented in supplementary table 1.

Supplementary Table 1: Description of seven-step transitional care pathway at the Jeroen Pit Huis

| **Location** | **Step** | **Description** |
| --- | --- | --- |
| Hospital | Step 1: Screening patients | The hospital team initiates early discharge planning, identifies potential candidates for the Jeroen Pit Huis transitional care unit (TCU), and consults the TCU pediatrician or nurse. Standardized screening ensures that a comprehensive initial care plan is in place before transfer to the TCU. |
| Jeroen Pit Huis | Step 2: Adjustment at TCU | Families acclimate to the new setting and recover from hospital-related stress. Transition goals are refined, and an initial care plan is further developed in collaboration with the family and the TCU team. |
|  | Step 3: Training at TCU | Parents gradually take on caregiving responsibilities while balancing their own well-being. Together with the TCU team, they work on transition goals, adjust care when necessary, and build confidence in managing their child’s care and health-related decision-making. |
|  | Step 4: Finalizing at TCU | The team and family finalize the remaining aspects of the care plan, ensure that home care arrangements are in place, and establish the primary care team responsible for post-discharge support. The focus is on a safe and coordinated transition from the TCU to home. |
| Discharge home | Step 5: Discharge home | Parents assume full responsibility for their child’s care in a safe home environment. All necessary care and support services are in place, and parents know where to seek help if needed. |
| Home | Step 6: Stable at home | The family regains a sense of normalcy and control over their child’s care. Parents manage caregiving independently while maintaining access to necessary support networks and can easily reach primary care providers and other relevant professionals. |
|  | Step 7: Step up function | If needed, families can access additional support, including intensified home care, day care at the TCU, or planned/unplanned (re)admission. This step-up function provides a safety net for families facing unforeseen challenges. |

More information can be found online: [www.hetjeroenpithuis.nl](http://www.hetjeroenpithuis.nl)

More information about the scientific research within the Dutch Transitional Care Unit (TCU) Consortium can be found online: [www.tcuconsortium.nl](http://www.tcuconsortium.nl)

*Reference supplementary 1*

1. Handboek JPH [cited 2023. Available from: <https://hetjeroenpithuis.nl/voor-ouders/informatie-over-het-jeroen-pit-huis/>.

2. van de Riet L, Alsem MW, Beijneveld RS, van Woensel JB, Van Karnebeek CD. Delineating family needs in the transition from hospital to home for children with medical complexity: part 2, a phenomenological study. Orphanet Journal of Rare Diseases. 2023.

**Supplementary 2: Standards for Reporting Qualitative Research (SRQR)***

|  | | **Page/line no(s).** |  |
| --- | --- | --- | --- |
| **Title and abstract** |  | | |
| **Title** - Concise description of the nature and topic of the study Identifying the study as qualitative or indicating the approach (e.g., ethnography, grounded theory) or data collection methods (e.g., interview, focus group) is recommended | | P1 (lines 1-2) |  |
| **Abstract** - Summary of key elements of the study using the abstract format of the intended publication; typically includes background, purpose, methods, results, and conclusions | | P1 (lines 4 – 27) |  |
|  | |  |  |
| **Introduction** |  | | |
| **Problem formulation** - Description and significance of the problem/phenomenon studied; review of relevant theory and empirical work; problem statement | | P3/4 (lines 48-90) |  |
| **Purpose or research questio**n - Purpose of the study and specific objectives or questions | | P4 (lines 85-90) |  |
|  | |  |  |
| **Methods** |  | | |
| **Qualitative approach and research paradigm** - Qualitative approach (e.g., ethnography, grounded theory, case study, phenomenology, narrative research) and guiding theory if appropriate; identifying the research paradigm (e.g., postpositivist, constructivist/ interpretivist) is also recommended; rationale** | | P4 (lines 93-96) |  |
| **Researcher characteristics and reflexivity** - Researchers’ characteristics that may influence the research, including personal attributes, qualifications/experience, relationship with participants, assumptions, and/or presuppositions; potential or actual interaction between researchers’ characteristics and the research questions, approach, methods, results, and/or transferability | | P6 (lines 147-152) |  |
| **Context** - Setting/site and salient contextual factors; rationale** | | P6-7 (lines 98-128) |  |
| **Sampling strategy** - How and why research participants, documents, or events were selected; criteria for deciding when no further sampling was necessary (e.g., sampling saturation); rationale** | | P6 (lines 137-143) |  |
| **Ethical issues pertaining to human subjects** - Documentation of approval by an appropriate ethics review board and participant consent, or explanation for lack thereof; other confidentiality and data security issues | | P8 (lines 184-187) |  |
| **Data collection methods** - Types of data collected; details of data collection procedures including (as appropriate) start and stop dates of data collection and analysis, iterative process, triangulation of sources/methods, and modification of procedures in response to evolving study findings; rationale** | | P6-7 (lines 145-166) |  |
| **Data collection instruments and technologies** - Description of instruments (e.g., interview guides, questionnaires) and devices (e.g., audio recorders) used for data collection; if/how the instrument(s) changed over the course of the study | | P6-7 (lines 145-166) |  |
| **Units of study** - Number and relevant characteristics of participants, documents, or events included in the study; level of participation (could be reported in results) | | P8 (lines 190-197) |  |
| **Data processing** - Methods for processing data prior to and during analysis, including transcription, data entry, data management and security, verification of data integrity, data coding, and anonymization/de-identification of excerpts | | P7-8 (lines 169-182) |  |
| **Data analysis** - Process by which inferences, themes, etc., were identified and developed, including the researchers involved in data analysis; usually references a specific paradigm or approach; rationale** | | P7-8 (lines 169-182) |  |
| **Techniques to enhance trustworthiness** - Techniques to enhance trustworthiness and credibility of data analysis (e.g., member checking, audit trail, triangulation); rationale** | | P7-8 (lines 169-182) |  |
|  | |  |  |
| **Results/findings** |  | | |
| **Synthesis and interpretation** - Main findings (e.g., interpretations, inferences, and themes); might include development of a theory or model, or integration with prior research or theory | | Page 8- 13 (lines 199-313) |  |
| **Links to empirical data** - Evidence (e.g., quotes, field notes, text excerpts, photographs) to substantiate analytic findings | | Table 3 |  |
|  | |  |  |
| **Discussion** |  | | |
| **Integration with prior work, implications, transferability, and contribution(s) to the field -** Short summary of main findings; explanation of how findings and conclusions connect to, support, elaborate on, or challenge conclusions of earlier scholarship; discussion of scope of application/generalizability; identification of unique contribution(s) to scholarship in a discipline or field | | Page 13 -19 (lines 315-463) |  |
| **Limitations** - Trustworthiness and limitations of findings | | Page 17 (lines 425-431) |  |
|  | |  |  |
| **Other** |  | | |
| **Conflicts of interest** - Potential sources of influence or perceived influence on study conduct and conclusions; how these were managed | | N/A |  |
| **Funding** - Sources of funding and other support; role of funders in data collection, interpretation, and reporting | | Title page |  |
|  | |  |  |
|  | |  |  |

**Supplementary 3: Interview guide**

| Introduction | Introduction: introducing the researchers and explaining the purpose of the study.  Research is voluntary (allowed to stop at any time, only information that participant wants to share) and confidential (safe storage of data, cannot be traced back to individual).  Approval for recording and for anonymized use of data in publication.  Timing of the interview. |
| --- | --- |
| (1) Introduction  *(Start audio recording)* | Can you introduce yourself and your family?  *Prompts:*  *How many children are in the family?*  *Does anyone else besides you take care of your child?* |
| (2) Hospital stay and transition to JPH | Your child has been admitted to the hospital. Can you tell me a little about how you experienced this hospital admission?  *Prompts:*  *Can you tell me about why your child was admitted to the hospital?*  *How long was your child admitted to the hospital?*  *At what point did you first learn about the JPH?*  *How did the transition from the hospital to the JPH go for you?*  *Looking back, is there anything that could have been better in this transition?* |
| (3) Jeroen Pit Huis stay | Can you tell me a little about how you experienced the transition from the hospital to the JPH?  *Prompts:*  *How were the first few days?*  *Was it clear what your goals were for your stay in the JPH?*  *How was the middle part of your stay?*  *What feelings did you experience throughout your stay? What helped you feel supported and what felt difficult?*  *How did the final period play out right before you returned home?*  *Did you feel adequately prepared to go home?* |
| (4) Home | And after the stay at the JPH, you went home. Can you share your experience of the transition from JPH to home?  *Prompts:*  *Which assistance contributed the most to a smooth transition home?*  *Was it clear how the care would be arranged? Or were there things that were unclear or difficult?*  *Did you have any contact with the JPH after discharge?* |
| (5) Unmet needs and points for improvement | Now that you’re back home, looking back, do you generally feel that the JPH has added value to your family?  *Prompts:*  *Do you have one or more tips for the JPH?* *For example, things that were missing or could be improved?*  *Can you highlight what you considered the best aspect of your stay at the JPH?* |
| End  *(Stop audio recording)* | We are nearing the end of the interview. Are there things we haven't discussed that you would like to add?  How did you experience this interview?  Thank you for participating and information about follow-up  Conducting a demographic data questionnaire |
